# *N*-oleoylethanolamide treatment of lymphoblasts deficient in Tafazzin improves cell growth and mitochondrial morphology and dynamics

**DOI:** 10.1038/s41598-022-13463-z

**Published:** 2022-06-08

**Authors:** John Z. Chan, Maria F. Fernandes, Klaudia E. Steckel, Ryan M. Bradley, Ashkan Hashemi, Mishi R. Groh, German Sciaini, Ken D. Stark, Robin E. Duncan

**Affiliations:** 1 Department of Kinesiology and Health Sciences, BMH 1044, Faculty of Health, University of Waterloo, Waterloo, N2L 3G1 Canada; 2 Department of Biology, Faculty of Science, University of Waterloo, Waterloo, N2L 3G1 Canada; 3 Department of Chemistry, Faculty of Science, University of Waterloo, Waterloo, N2L 3G1 Canada

**Keywords:** Mechanisms of disease, Metabolic disorders, Cellular imaging

## Abstract

Barth syndrome (BTHS) is caused by mutations in the *TAZ* gene encoding the cardiolipin remodeling enzyme, Tafazzin. The study objective was to quantitatively examine growth characteristics and mitochondrial morphology of transformed lymphoblast cell lines derived from five patients with BTHS relative to five healthy controls, as well as the therapeutic potential of oleoylethanolamide (OEA) and linoleoylethanolamide (LEA). These bioactive lipids both activate PPARα, which may be therapeutic. BTHS lymphoblasts grew more slowly than controls, suggesting lymphopenia merits clinical investigation. Treatment of BTHS lymphoblasts with OEA, but not LEA, significantly restored mitochondrial membrane potential, as well as colony growth in all BTHS lymphoblast lines, although a full growth rescue was not achieved. Quantification analysis of electron micrographs from three BTHS and healthy lymphoblast donors indicated similar numbers of mitochondria per cell, but lower average cristae length per mitochondrion, and higher mitochondrial density. Additionally, BTHS lymphoblasts had larger mitochondria, and a higher percentage of abnormally large mitochondria (> 1 μm^2^) than healthy controls. Notably, OEA treatment significantly restored mitochondrial size, without affecting density or cristae lengths. Cardiolipin total content, relative linoleic acid content and monolysocardiolipin:cardiolipin ratios were not improved by OEA, indicating that effects on growth, and mitochondrial morphology and function, occurred without resolving this deficit. However, immunoblotting showed higher levels of OPA1, a biomarker for mitochondrial fusion, in BTHS lymphoblasts, which was attenuated by OEA treatment, implicating altered mitochondrial dynamics in the pathology and treatment of BTHS.

## Introduction

Barth Syndrome (BTHS), first reported by Dr. Peter Barth in 1983, is a rare X-linked genetic disorder caused by mutations in the *TAZ* gene that encodes for the mitochondrial enzyme Tafazzin^1,2^. Tafazzin functions in the remodelling of nascent cardiolipin, a process that involves sequential de-acylation and re-acylation, in which the fatty acyl chains that were incorporated in de novo synthesis are replaced primarily with 18-carbon fatty acids^3,4^. Since different tissues require different fatty acyl profiles to maintain proper functioning, this process is necessary to generate the specialized, mature forms of cardiolipin needed to match cell-specific demands^4^. A variety of mutations in the *TAZ* gene have been identified, but the common result of Tafazzin enzyme dysfunction is an accumulation of monolysocardiolipin (MLCL), and a concurrent reduction in mature cardiolipin levels^1,5^. This results in the disruption of mitochondrial ultrastructure, destabilization of the electron transport chain proteins embedded therein, mitochondrial swelling, and impaired oxidative phosphorylation^6–8^. As a result, BTHS patients with *TAZ* deficiencies usually manifest symptoms of dilated cardiomyopathy within the first year of life, leading to progressive heart failure^9^. BTHS is also characterized by neutropenia, skeletal myopathy, 3-methylglutaconic aciduria, and some varying degree of cognitive impairment, although many individuals with BTHS complete advance study^9–11^.

Within the recent decade, much of the clinical data on immune aspects of BTHS have focused on the innate immune system, with studies examining mechanisms behind the decrease in neutrophil count in BTHS patients leading to neutropenia^11–15^. However, little is known about adaptive immunity in BTHS, and whether or not BTHS patients also manifest symptoms of lymphopenia. In a recent study by Corrado et al., the peripheral blood mononuclear cells from five individual BTHS patients with a mean age of 13 years were examined^16^. It was discovered that patients with BTHS had lower percentages of CD8^+^ T cells and CD8^+^ T cells producing interferon-γ compared to healthy donors^16^. This finding suggests that the adaptive immune system may also be affected in BTHS, and merits further examination.

In the current work, we have utilized Epstein-Barr virus-transformed, patient-derived lymphoblasts from the Coriell Institute for Medical Research, National Institute of General Medical Sciences (NIGMS)—human genetic cell repository^8,17–19^. Lymphoblasts are immature precursor cells that originate in the bone marrow and can give rise to lymphocytes of both the T and B lineage^20^. Previous studies by Acehan et al*.* and Gonzalvez et al*.* have examined the mitochondrial morphology of some of these lymphoblast lines derived from different BTHS and healthy donors (hereafter referred to as BTHS lymphoblasts and healthy lymphoblasts, respectively)^8,17^. As expected, the mitochondria from healthy donors exhibited a characteristic parallel cristae structure, which connected from the inner boundary membrane, and often spanned the entire length of the organelle^8,17^, but in BTHS lymphoblasts, the morphology of the mitochondria was aberrant. In particular, swollen mitochondria were observed, lacking proper alignment of cristae that were often disconnected from the peripheral membrane, giving an ‘onion-shaped’ or ‘honey-comb shaped’ morphology^8,17^. In addition, there appeared to be an increased frequency of enlarged mitochondria. While the alternations in mitochondrial cristae associated with BTHS are thought to be related to the deficiency of cardiolipin, the mechanism underlying the increase in mitochondrial size is not yet understood^8,21^. Additionally, the expansion of BTHS lymphoblast lines in culture has not yet been measured. Studies on the growth characteristics of BTHS lymphoblast lines from different donors could provide valuable information on the possibility of latent lymphopenia in this disease.

The first objective of this study was to characterize the cell number expansion patterns of five donor-derived BTHS lymphoblast and healthy control lines, and related effects of treatment with the bioactive lipids oleoylethanolamide (OEA) and linoleoylethanolamide (LEA). OEA and LEA belong to a group of *N*-acylethanolamines (NAEs), comprised of a fatty acyl chain attached to an ethanolamine group^22^. Both are synthesized endogenously^22^, but can also be derived from the diet^23^ and supplements^24^, and are activating ligands for peroxisome proliferator-activated receptor alpha (PPARα)^25^. PPARα stimulation by the drug Bezafibrate has previously been shown to ameliorate the cardiomyopathy symptoms of *TAZ* knockdown mice, and is currently the subject of a clinical trial^26–28^, and therefore PPARα activating compounds are of clinical interest in this disease. Despite these similarities, however, only OEA demonstrated a significant effect on the growth of BTHS lymphoblasts, and this was not observed with LEA. Therefore, follow-up studies were limited to this endocannabinoid-like compound. In our second and third objectives, effects of OEA on the mitochondrial morphology, function, and cardiolipin content of BTHS and healthy lymphoblasts were assessed, as well as on the expression of proteins involved in regulating mitochondrial dynamics.

## Results

### Treatment with OEA partially rescued a deficit in BTHS lymphoblast colony growth

**Figure 1:**
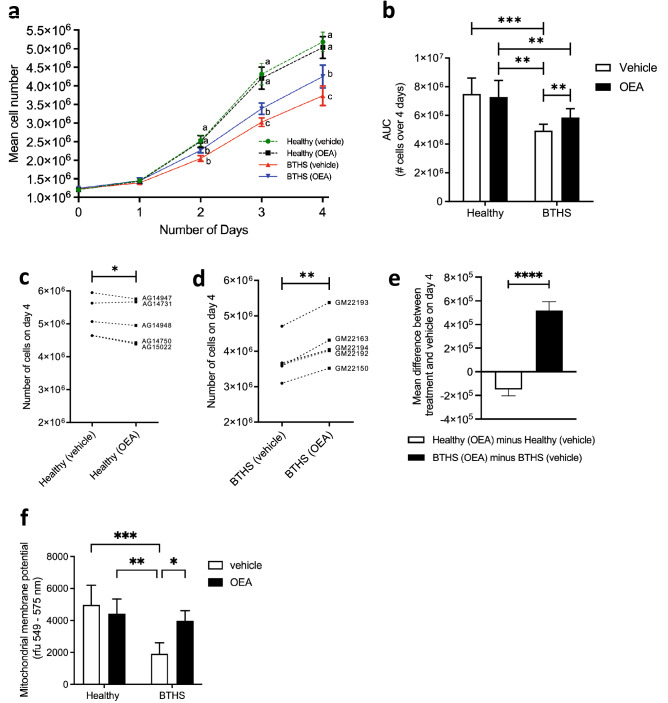
BTHS lymphoblasts exhibit impaired growth that is partially rescued by OEA treatment. Mean daily cell counts are shown from cultures of five healthy and five BTHS lymphoblast lines treated with either vehicle control (0.1% (v/v) ethanol) or OEA [1 µM] for 4 days (**a**), with accompanying area-under-the-curve analysis (**b**). The individual cell line responses to 1 µM OEA treatment are represented as cell counts on day 4 (vehicle versus OEA) for each of the five healthy (**c**) and five BTHS (**d**) lines. A comparison of the relative effect of treatment in healthy versus BTHS lines on mean cell number is also shown (**e**). Effects of OEA on mitochondrial membrane potential in all cell lines were measured (**f**). Data are means ± S.E.M; *n* = 5. ^abc^Groups with different letters are significantly different at the same timepoint, P < 0.05. *P < 0.05, **P < 0.01, ***P < 0.001, ****P < 0.0001.

In our initial work with the lymphoblast cultures, we observed that the BTHS lines appeared to expand at a slower rate than the healthy lines. To the best of our knowledge, there have been no reports of reduced colony expansion in prior studies of BTHS lymphoblast lines^8,17–19,29^, or reports of lymphopenia in patients with BTHS. We therefore conducted a careful measurement of the expansion of BTHS and healthy lymphoblast lines in culture (Fig. 1a). As expected, after an initial lag-phase on day 1, all cell lines increased in number daily. Repeated-measures ANOVA revealed statistically significant differences between healthy and BTHS lymphoblast numbers within treatments, with healthy lines having higher cell numbers than BTHS lines already by day 2, regardless of treatment. This growth deficit was substantial, with the cell number difference between vehicle-treated BTHS and vehicle-treated healthy lymphoblast reaching as high as ~ 1.5 million cells by day 4 (P < 0.0001). However, by day 3, while healthy, vehicle-treated lymphoblast numbers were still higher than vehicle-treated BTHS lymphoblast numbers, the number of BTHS lymphoblasts in OEA-treated cultures was significantly greater than the numbers in corresponding BTHS cultures grown only in vehicle. Although promising, this rescue was only partial, since the numbers of cells in the OEA-treated BTHS cultures were still significantly lower than in OEA-treated healthy cell cultures. A summary of differences in the cell number growth curves can be seen in Fig. 1b, where area-under-the-curve (AUC) has been analyzed for all four groups.

Each healthy and BTHS lymphoblast line was derived from a distinct human donor, and consistent differences were noted among these lines, such that after equal seeding of ~ 1.2 million cells, total cell number on day four varied by upwards of ~ 1.3 million cells among healthy lymphoblast cell lines, and ~ 1.6 million cells among BTHS lymphoblast cell lines. To look at individual patterns of response among healthy (Fig. 1c) and BTHS (Fig. 1d) lymphoblast lines, we graphed the number of cells on day 4 in untreated versus treated lines, and performed a pairwise comparison. Among the healthy lymphoblast lines, total cell number was slightly lower on day 4 in four out of five lines treated with OEA versus vehicle, and this relative decrease was statistically significant when analyzed by paired Student’s t-test. Conversely, there was a greater number of cells following OEA treatment in all five BTHS lymphoblast lines on day 4, compared to vehicle alone, indicating a consistent, positive response to OEA. The relative differences in mean response to OEA treatment between healthy donor lines and BTHS donor lines is illustrated in Fig. 1e, which shows that on average, healthy lines treated with OEA had ~ 1.5 × 10^5^ fewer cells than their vehicle-treated controls, while BTHS lines treated with OEA had ~ 5 × 10^5^ more cells than their vehicle-treated controls, and the difference between the responses of healthy and BTHS lines was highly statistically significant. Mitochondrial membrane potentials were analyzed as a measure of mitochondrial function after 4 days of cell treatment with OEA or vehicle, and found to be significantly lower in vehicle-treated BTHS lymphoblasts compared to healthy controls treated either with vehicle or OEA. However, this measure was significantly restored in BTHS cells by OEA (Fig. 1f), suggesting that better mitochondrial function may contribute to the growth benefit of OEA in BTHS lymphoblasts.

### Treatment with LEA did not rescue a deficit in BTHS lymphoblast colony growth

**Figure 2:**
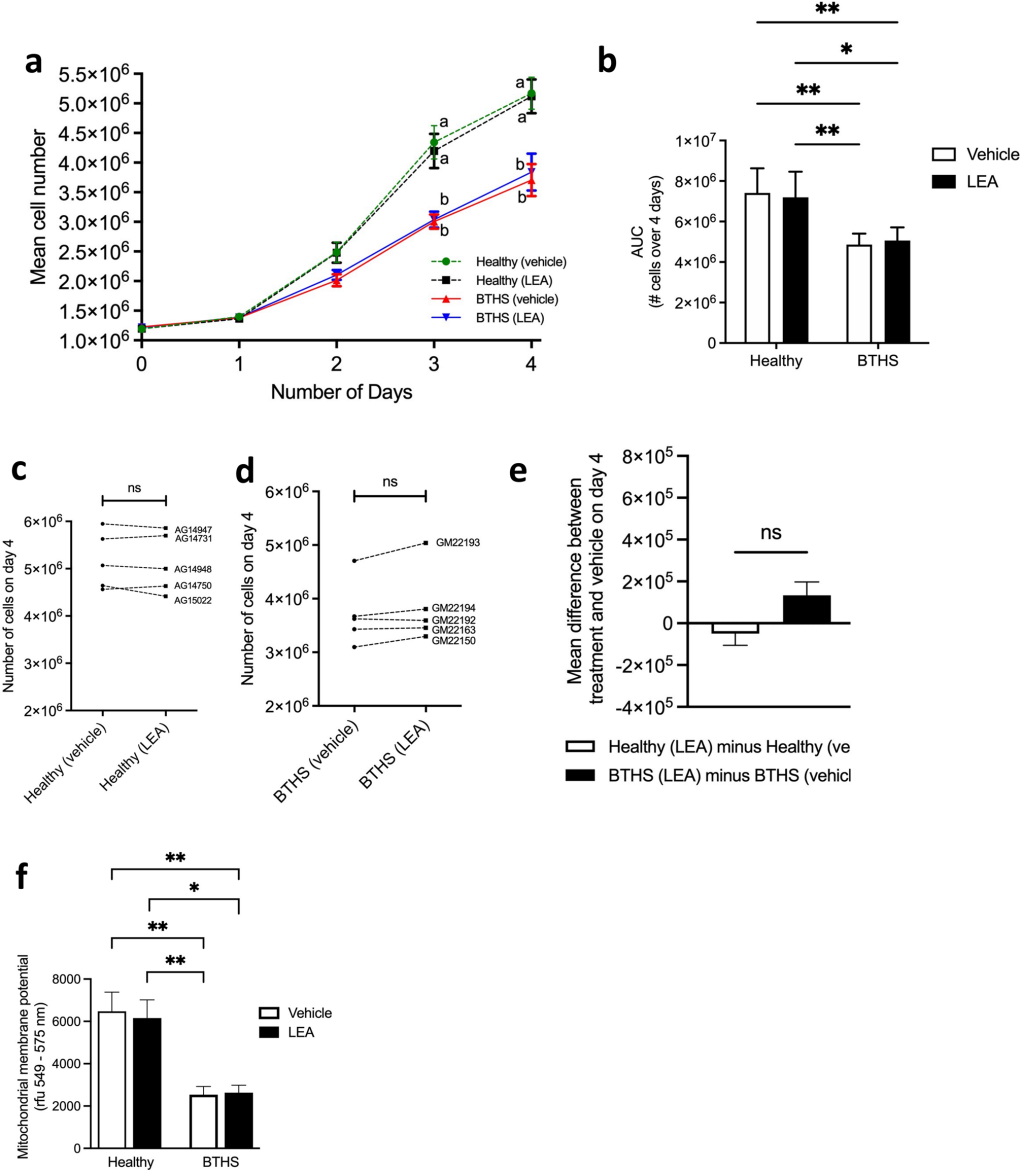
BTHS lymphoblasts exhibit impaired growth that is not rescued by LEA treatment. Mean daily cell counts from cultures of five healthy and five BTHS lymphoblast lines treated with either vehicle control (0.1% (v/v) ethanol) or LEA [1 µM] for 4 days (**a**). Area-under-the-curve (AUC) analysis (**b**). Individual cell line counts on day 4 (vehicle versus 1 µM LEA) for each of the healthy lines (**c**) and BTHS lines (**d**). The relative effect of treatment in healthy versus BTHS lines on mean cell number is shown (**e**). Effect of LEA on mitochondrial membrane potential in healthy and BTHS cell lines (**f**). Data are means ± S.E.M; *n* = 5. ^ab^Groups with different letters are significantly different at the same timepoint, P < 0.05. *P < 0.05, **P < 0.01, *ns* not significant.

Studies with LEA indicated a lack of significant effect of the compound on cell growth in either healthy or BTHS cell lines. Repeated measures ANOVA detected a statistically significant effect of cell line but not LEA treatment. Significant differences between mean numbers of healthy and BTHS cells were observed by day 2, and this difference remained throughout the timepoints measured but was not altered by LEA treatment at any timepoint (Fig. 2a), or overall (Fig. 2b). Quantitation of lymphoblast numbers on day 4 in cells treated with LEA indicated no significant effect in either healthy (Fig. 2c) or BTHS (Fig. 2d) lines. In healthy lines, the mean difference observed was a reduction in cell number by ~ 0.5 × 10^5^ cells with LEA treatment, whereas the mean difference in BTHS lines was an increase in cell number of ~ 1.3 × 10^5^ cells (Fig. 2e), although these values were not statistically significantly different. Similarly, while the mean mitochondrial membrane potential was lower in BTHS cell lines compared to healthy lines, this difference was not significantly affected by LEA treatment (Fig. 2f). Thus, additional experiments examining mitochondrial morphology and dynamics were performed using only OEA.

### Treatment with OEA did not rescue cardiolipin total content in BTHS lymphoblasts

**Figure 3:**
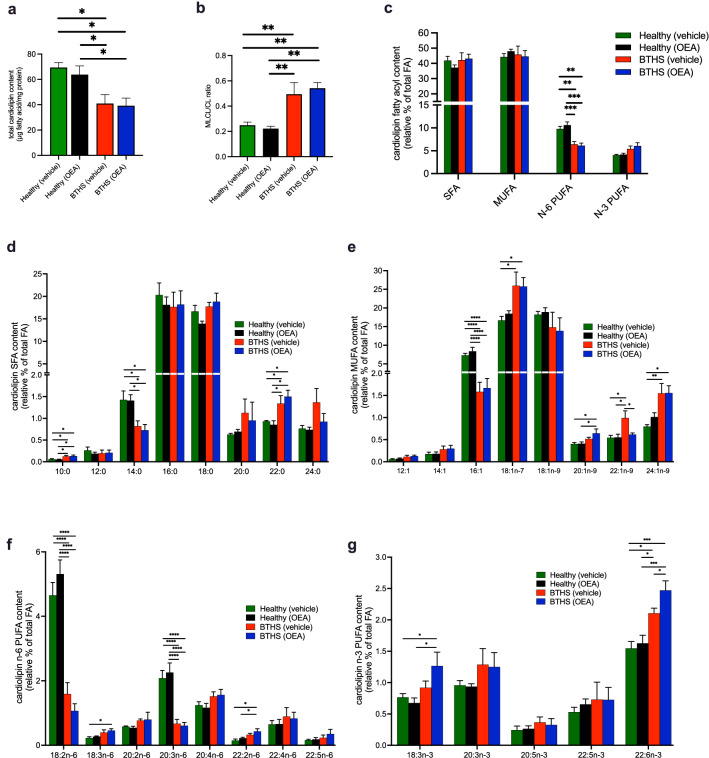
OEA does not restore total cardiolipin content or MLCL:cardiolipin ratio in BTHS lymphoblasts. Total cardiolipin content (**a**), MLCL:cardiolipin ratio (**b**), cardiolipin fatty acyl (FA) content by major categories (**c**), and cardiolipin relative contents of saturated (SFA) (**d**), monounsaturated (MUFA) (**e**), n-3 polyunsaturated (N-3 PUFA) (**f**) and n-6 polyunsaturated (N-6 PUFA) (**g**) fatty acyl species. Data are means ± S.E.M; *n* = 5. *P < 0.05, **P < 0.01, ***P < 0.001, ****P < 0.0001.

There was no effect of 4 days of OEA treatment on total cardiolipin content (Fig. 3a), MLCL:cardiolipin ratio (Fig. 3b), or relative fatty acyl composition of cardiolipin (Fig. 3c–g) in healthy cells. As expected, the total cardiolipin content was significantly lower in vehicle-treated BTHS lymphoblasts compared to healthy control cells (either treated or untreated) (Fig. 3a) and the MLCL:cardiolipin ratio was significantly higher (Fig. 3b). Also as expected, the total n-6 polyunsaturated fatty acid (PUFA) content was significantly lower, while significant differences in total saturated fatty acids (SFA), monounsaturated fatty acids (MUFA), and n-3 PUFA were not different (Fig. 3c). Surprisingly, however, despite the improvement in cell growth (Fig. 1a–e) and mitochondrial membrane potential (Fig. 1f) that was observed in BTHS lymphoblasts following OEA treatment, there was no significant improvement in total cardiolipin content or MLCL:cardiolipin ratio (Fig. 3a,b), and no significant increase in total n-6 PUFA (Fig. 3c). The only specific difference between vehicle-treated BTHS cells and OEA-treated BTHS cells was a significant decrease in the cardiolipin relative content of 22:1 n-9, erucic acid and a significant increase in 22:6n-3 (i.e. docosahexaenoic acid).

Although the relative content of total SFA, total MUFA and total n-3 PUFA were not significantly different across groups, some differences in the abundance of specific fatty acyl species from these groups, as well as n-6 PUFA, were evident (Fig. 3d–g). Regardless of treatment, among SFA species, levels of C10:0 and C22:0 were significantly higher in BTHS lines compared to healthy lines, and levels of C14:0 were significantly lower (Fig. 3d). Among MUFA species, C16:1n-7 was significantly lower in BTHS lines than in healthy lines, also irrespective of treatment, while C18:1n-7 and C24:1n-9 levels in BTHS lines were significantly higher than in vehicle-treated healthy controls, and C20:1n-9 levels in OEA-treated BTHS cells were higher than in either healthy control group (Fig. 3e). In addition to the expected lower relative content of 18:2n-6 (linoleic acid) in BTHS lymphoblasts compared to healthy controls, there was also a lower content of 20:3n-6, and both effects were irrespective of treatments (Fig. 3f). Among n-3 PUFA, there was some evidence of higher 18:3n-3 (linolenic acid) content in cardiolipin from BTHS cells treated with OEA, and higher levels of 22:6n-3 in BTHS lymphoblasts under either treatment condition compared to healthy controls (Fig. 3g).

### Treatment with OEA improved the morphology of mitochondria in BTHS lymphoblasts

**Figure 4:**
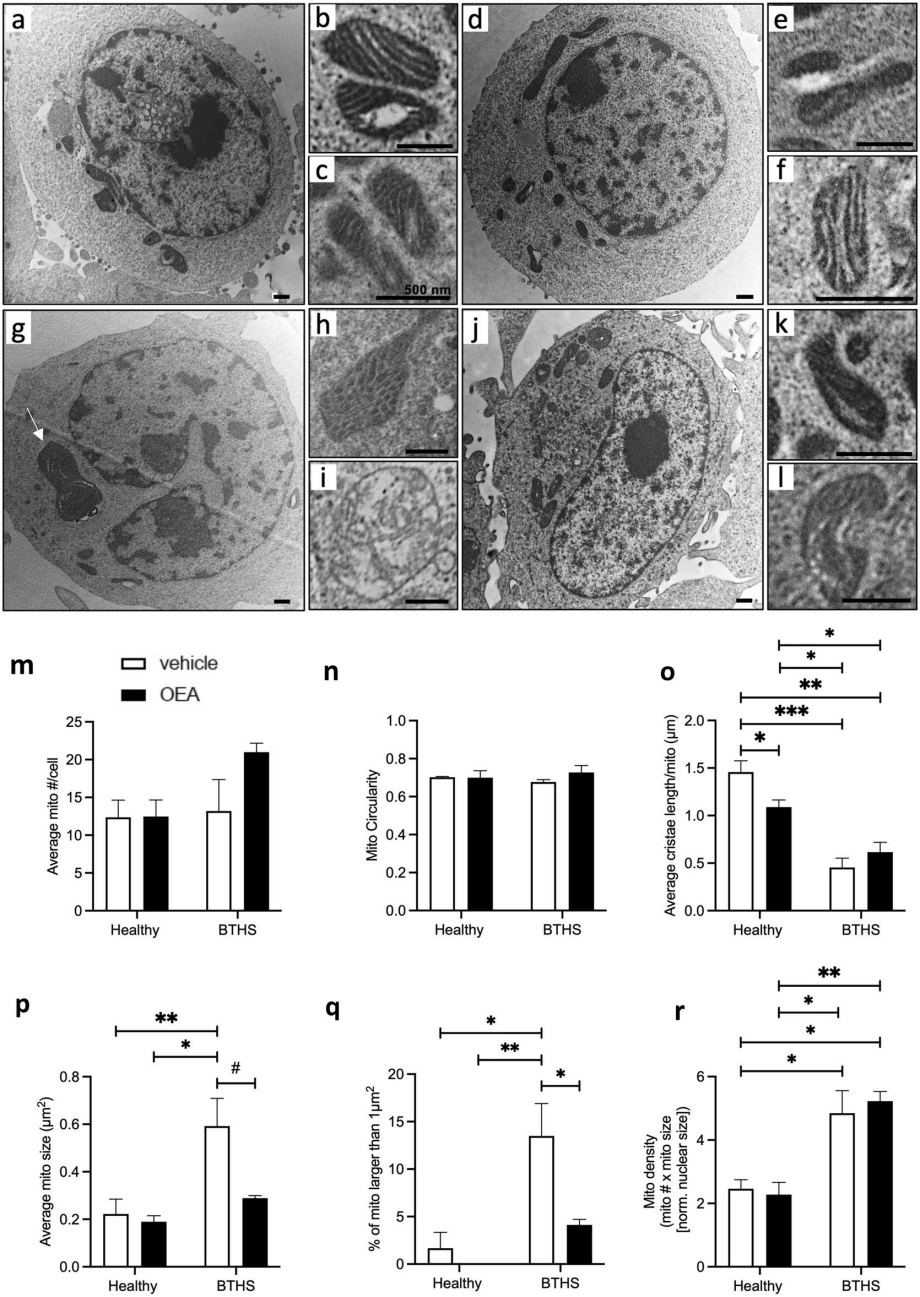
BTHS lymphoblasts have larger mitochondria that are reduced in size to healthy lymphoblast levels by OEA treatment**.** Representative whole cell and subcellular mitochondrial images are shown for vehicle-treated healthy lymphoblasts (**a–c**), 1 µM OEA-treated healthy lymphoblasts (**d–f**), vehicle-treated BTHS lymphoblasts (**g–i**), and 1 µM OEA-treated BTHS lymphoblasts (**j–l**). The white arrow in (**g**) shows a characteristic, atypically enlarged mitochondrion from a BTHS lymphoblast, while the image in (**h**) shows a mitochondrion with a ‘bubbly’ appearance, and the image in (**i**) shows a mitochondrion with an ‘onion-like’ appearance. Quantitative measures were derived from images produced from three of the BTHS and healthy lymphoblast lines after either vehicle treatment or treatment with 1 µM OEA for 4 days. The average number of mitochondria per cell (normalized to nuclear area to account for differences in cross-sectional area) is shown (**m**). Measures of mitochondrial morphology, including mitochondrial circularity (**n**), average total parallel cristae length per mitochondrion (**o**), average mitochondrial size (**p**), and the prevalence of atypically large mitochondria (**q**) are also reported. Scale bars = 500 nm. Data are means ± S.E.M; *n* = 3. ^#^P = 0.06, *P < 0.05, **P < 0.01, ***P < 0.001.

Differences in mitochondrial morphology were compared between three BTHS (GM22192, GM22163, GM22150) and three healthy (AG15022, AG14948, and AG14750) lymphoblast lines. Representative micrographs are shown in Fig. 4a–l, with quantitation graphed in Fig. 4m–r. A cross-section of a healthy lymphoblast is shown in Fig. 4a–c showing representative healthy mitochondria with visibly distinct cristae. Figure 4d shows a cross-section of a healthy lymphoblast treated with OEA, with inset representative images of mitochondria also showing normal striations (Fig. 4e,f), essentially unchanged by OEA treatment. Figure 4g shows a cross-section of a BTHS lymphoblast, where a characteristically enlarged mitochondrion is visible in the cytoplasm. Figure 4h,i show additional images of abnormal mitochondria, with cristae that form ‘bubbly’ or ‘onion-like’ structures, respectively, rather than parallel cristae. Conversely, Fig. 4j shows a cross-section of a BTHS lymphoblast, while Fig. 4k,l illustrate the more normal appearance of mitochondria in BTHS lymphoblasts treated with OEA.

No statistically significant differences were observed in the average number of mitochondria per cell, with counts normalized to nuclear area to account for differences in the cross-sectional plane analyzed (Fig. 4m), or in mitochondrial circularity (Fig. 4n) between BTHS and healthy lymphoblasts treated with OEA or vehicle. The average parallel cristae length per mitochondrion was reduced by 25% in healthy lymphoblasts by OEA treatment (Fig. 4o). However, the average parallel cristae length per mitochondrion was already 69% lower in vehicle-treated BTHS lymphoblasts compared to vehicle-treated healthy lymphoblast controls (P < 0.05), and remained significantly lower in BTHS lymphoblasts compared to healthy lymphoblasts, and this measure was not significantly increased by treatment of BTHS lymphoblasts with OEA (Fig. 4o). Conversely, differences between healthy and BTHS lymphoblast lines were evident when mitochondrial size was evaluated, along with BTHS-specific beneficial effects of OEA treatment. Mitochondria in vehicle-treated BTHS lymphoblasts were, on average, 2.66-fold larger than mitochondria in vehicle-treated healthy lymphoblasts, and 3.12-fold larger than mitochondria in OEA-treated healthy lymphoblasts (Fig. 4p). Treatment of BTHS lymphoblasts with OEA reduced average mitochondrial size to measures that did not differ significantly from measures in healthy lymphoblasts and approached a significant reduction from vehicle-treated BTHS lymphoblasts (P = 0.06). Differences in mitochondrial size are highlighted by the use of a threshold measure. Mitochondria with cross-sectional areas larger than 1 µm^2^ were found in one of the three vehicle-treated healthy lymphoblast lines evaluated, but were not seen in any of the healthy lymphoblast lines treated with OEA (Fig. 4q). In contrast, all BTHS lines, regardless of treatment, had mitochondria with cross-sectional areas > 1 µm^2^. However, these abnormally large mitochondria were almost 70% less common when cells were treated with OEA, constituting 13.5% of mitochondria in vehicle-treated BTHS lines, versus only 4.1% of mitochondria in OEA-treated BTHS lines (P < 0.05). Mitochondrial density, calculated as the number of mitochondria per cell (normalized to nuclear area) times the average mitochondrial size, was significantly greater in BTHS lines compared to healthy lines, but did not differ by treatment (Fig. 4r).

### OEA decreased OPA1 expression in BTHS lymphoblasts

Analysis of images in Fig. 4 indicated that one of the most consistent quantitative differences between healthy and BTHS lymphoblasts was the occurrence of atypically large mitochondria. This was also the difference that was most affected by OEA treatment. While the average cristae length per mitochondrion was significantly lower in BTHS compared to healthy cell lines, this measure was not significantly altered in BTHS lymphoblasts by OEA treatment, whereas measures of mitochondrial size showed promising decreases towards healthy levels. This suggested that factors involved in mitochondrial size regulation should be investigated.

We used immunoblotting to determine the expression levels of several dynamin-like GTPases that are involved in mitochondrial dynamics regulation, and full images can be seen in Supplementary Figs. S1–S6. Mitochondria are highly dynamic organelles that continuously undergo both fission and fusion events to alter shape and size in response to demands from the cellular environment^30^. Fusion of the inner mitochondrial membrane is mediated by the optic atrophy 1 (OPA1) protein, and fusion of the outer mitochondrial membrane is regulated by the mitofusins (MFN1 and MFN2)^30^. Dynamin-related protein 1 (DRP1) is the central regulator of fission, and is controlled by post-translational modifications^30^. For example, the phosphorylation of DRP1 at Ser616 (pDRP1(Ser616)) by MAPK stimulates fission, whereas the phosphorylation of DRP1at Ser637 (pDRP1(Ser637)) inhibits its GTPase activity, and thus inhibits fission^31,32^.

**Figure 5:**
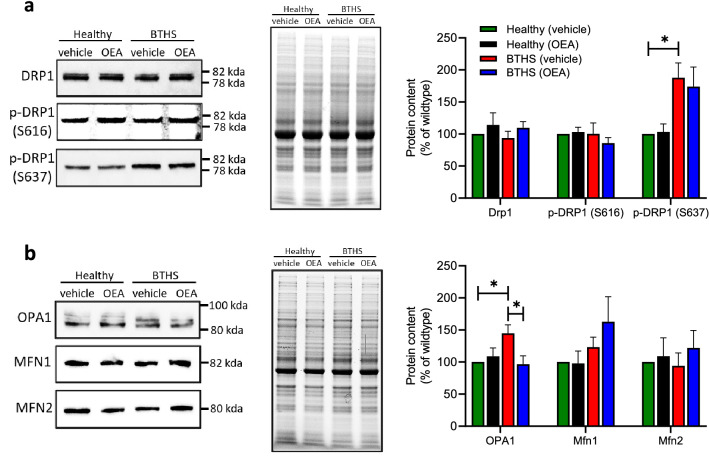
OEA treatment attenuated the elevated expression levels of OPA1 in BTHS lymphoblasts. Representative immunoblots (left panel), images of total protein loading (middle panel), and quantification (right panel) of proteins involved in mitochondrial fission (**a**) and fusion (**b**) are shown. Quantification was determined by Image Lab software (Bio-Rad Canada) and shown normalized to total protein. Uncropped images are presented in Supplementary Figs. S1–S6. Data are means ± S.E.M; *n* = 5. *P < 0.05.

Immunoblot analysis indicated no differences in total levels of DRP1 or pDRP1(Ser616) between BTHS and healthy lymphoblasts, regardless of treatment (Fig. 5a). In contrast, significantly higher phosphorylation levels of DRP1 at Ser637 were observed in BTHS lymphoblasts (P < 0.05) (Fig. 5a), suggesting possible fission inhibition as a factor in the larger mitochondria observed. However, OEA treatment had no effects on the regulation of DRP1 phosphorylation levels at Ser637 in either BTHS or healthy lymphoblasts, strongly suggesting that DRP1 phosphorylation regulation did not contribute to the OEA-mediated rescue of mitochondrial size that was observed. Interestingly, however, while total levels of the mitofusins were not different between healthy and BTHS lines, or affected by OEA treatment, expression levels of OPA1 in vehicle-treated BTHS lymphoblasts were 45% higher than healthy controls (P < 0.05), and essentially restored to healthy control levels by treatment with OEA (Fig. 5b). OEA had no significant effect on OPA1 expression in vehicle-treated healthy lymphoblasts.

## Discussion

A majority of the clinical data on immunity in BTHS has focused on the innate immune system, with studies suggesting that the decrease in neutrophil levels is likely due to increased clearance of neutrophils by tissue macrophages and/or increased myeloid progenitor apoptosis^11–15^. Conversely, little is known about effects of *TAZ* deficiency on the adaptive immune system, and whether some degree of lymphopenia is present in BTHS patients as well. Among circulating leukocytes, lymphocytes normally account for 20–30%, although this can rise as high as 40% during an infection^33,34^. Thus, an optimal time to investigate a putative lymphopenia is when patients are undergoing an active immune response, which can present clinical and ethical challenges. Additionally, the mechanistic study of lymphoid precursors (i.e. lymphoblasts), and lymphoid progenitor cells requires bone marrow aspiration, which can be invasive and dangerous, particularly for BTHS patients with clinical neutropenia^35^. To combat these challenges, we used Epstein-Barr virus-transformed lymphoblasts derived from BTHS patient B-lymphocytes, and transformed lymphoblasts from age, sex and race matched controls.

BTHS lymphoblasts have been studied previously^8,17–19,29^, and it is known that these lines can be expanded in culture for use in experiments. Although mitochondrial defects have previously been associated with growth deficiencies^36^, the slower growth rate of BTHS lymphoblast cultures relative to control lymphoblast cultures had not yet been reported. Our findings indicated differences in the number of cells between BTHS and control lymphoblast cultures that were already apparent by day 2, when a deficit in cell number was statistically significant. This difference became more pronounced on days 3 and 4. Although the mechanism underlying this effect was not determined in the current work, it is likely to involve a lower rate of cell proliferation, rather than a higher rate of cell loss through processes such as apoptosis. Proliferation is an energetically demanding process, and impaired energy production in BTHS is well documented^7,17,18^. Additionally, a critical role for *TAZ* in cell proliferation control, independent of cardiolipin production, has been demonstrated in several cell lines, and is of significant interest in understanding changes in malignancy^37–41^. In contrast, *TAZ-*deficiency in human BTHS and experimental models is associated with impaired apoptosis^17,29^, suggesting that a greater rate of cell loss through this pathway is not likely to be a significant mediator of lower cell numbers. Regardless of the mechanism involved, this finding is important since it suggests that some degree of lymphopenia may be present in BTHS patients and should be evaluated clinically.

OEA partially rescued BTHS lymphoblast numbers, while the related compound LEA did not have a significant effect*.* This difference may be related to variations in receptor-mediated signaling by these compounds, with OEA acting primarily via TRPV1, PPARα, and GPR119, and LEA primarily signaling through CB1, CB2, TRPV1, and PPARα^25,42–45^, although differences in affinity of the compounds for commonly shared receptors may also be a factor^46^. It is also possible that effects may occur independent of receptor-mediated activity. *N*-acylethanolamine hydrolysis results in the generation of free ethanolamine^47^, which exhibits growth-stimulatory effects in mammalian cells^48^, increasing hepatocyte cell proliferation rates both in vivo and in vitro^49^, and promoting mTOR signaling and mitochondrial function to increase the proliferative capacity of epithelial cells^50^. However, since OEA and LEA were provided at equimolar levels, cells are expected to derive similar concentrations of free ethanolamine from breakdown reactions, and therefore specific differences in the bioactivity and signaling activities of the two compounds is more likely responsible.

The improvement in BTHS lymphoblast cell numbers by OEA was associated with significantly restored mitochondrial membrane potential, indicating better mitochondrial function^51^. This was not apparent in cells treated with LEA. Moreover, the improvements in mitochondrial and cellular function with OEA treatment were not dependent on a restoration of total cardiolipin levels or the MLCL:cardiolipin ratio. These effects were also not dependent on a restoration of the relative linoleic acid content of cardiolipin, which was not significantly improved in BTHS lymphoblasts treated with OEA, or an obvious pattern of increase in any other fatty acyl species or major group. This finding is important, since it suggests that targeting other aspects of mitochondrial biology in BTHS, besides the deficiency in tetralinoleoyl cardiolipin, may have merit in the treatment of this syndrome.

In addition to improving growth characteristics of BTHS lymphoblasts, OEA treatment improved some aspects of mitochondrial morphology. Previous studies examining BTHS lymphoblasts have suggested there may be mitochondrial hyperproliferation^8,17,18^. This was based on observations of electron microscopy images, as well as analysis of mitochondrial copy number, which has been reported to be nearly threefold higher in BTHS lymphoblasts compared to healthy controls, and suggested a compensatory effect to maintain adequate ATP production despite reduced electron transport chain capacity^17,52^. In contrast, our analysis did not demonstrate a hyperproliferation of mitochondria, since the number of mitochondria per BTHS lymphoblast was similar to that recorded in healthy lymphoblasts. Rather, analysis of electron micrographs in the current study demonstrated an increase in mitochondrial density in BTHS lymphoblasts that was in agreement with the study by Gonzalvez et al.^17^. There could be several reasons for differences between our study and other studies in this regard. Although differences among individual cell lines utilized could be a factor, it is notable that the current study was the first to quantitate a series of mitochondrial parameters using imaging software and, importantly, reported data normalized to nuclear size, in order to account for the size of the cross-sectional images evaluated. The use of surrogate measures, such as mitochondrial DNA copy number, could also give the impression that the number of mitochondria had increased, when an enlargement of mitochondria may have occurred instead. The overall result from either approach would, however, be an indication that mitochondrial density had increased, which was in agreement between the present study, and prior reports. However, neither the average number of mitochondria per cell, nor the mitochondrial density, was significantly altered by OEA treatment.

Past studies have described BTHS lymphoblast mitochondria as containing fewer parallel cristae, and described the cristae structure of BTHS lymphoblasts as aberrant^8,17^. We employed a quantitative analysis and determined that average parallel cristae length per mitochondrion in BTHS lymphoblasts was less than a third of that measured in healthy lymphoblast controls. Aberrant mitochondria with ‘onion-ring’ shaped or ‘bubbly’ cristae were also evident. The reason for aberrant mitochondrial cristae structures in BTHS has not been fully elucidated. Decreases in total cardiolipin content, in conjunction with increases in MLCL, may be a factor affecting the curvature of the inner mitochondrial membrane^5,53^. In addition, shifts in the fatty acyl profile of cardiolipin from *TAZ*-deficient cells may also play a role in shaping the ultrastructure of the cristae, since remodeled cardiolipin has been shown to be vital for the proper alignment and organization of the cristae membrane, and cardiolipin from BTHS cells tends to have palmitoleic, oleic and linoleic acids replaced by higher levels of vaccenate, and the saturated fatty acids palmitate and stearate^18,21^. Interestingly, although OEA treatment significantly increased the number of BTHS lymphoblasts in culture, this was not related to a significant improvement in the average cristae length per mitochondrion, and there was no significant change in total cardiolipin content, and only minor changes in fatty acyl composition. Likewise, OEA did not significantly alter mitochondrial shape. Treatment with OEA did, however, significantly affect the size of mitochondria in BTHS lymphoblasts.

Mitochondria were, on average, close to 3-times larger in BTHS lymphoblasts than healthy lymphoblasts, and large mitochondria over 1 µm^2^ were more than 3 times as prevalent. Treatment with OEA reduced the size of BTHS lymphoblast mitochondria to measures that did not differ significantly from healthy lymphoblast levels. While an increased frequency of giant mitochondria in BTHS lymphoblasts has been reported by other groups, the reason for this change is unknown^8^. Gonzalvez et al. previously analyzed the total content of OPA1 oligomers in a different set of BTHS and healthy lymphoblast donors, and found that cardiolipin content did not play a major role in the incorporation of OPA1 into complex oligomeric structures^17^. To the best of our knowledge, however, no other studies have examined levels of mitochondrial dynamics regulators in BTHS. We therefore performed measures of major protein regulators involved in mitochondrial fission and fusion using cells treated either with vehicle or OEA, to examine differences between BTHS and healthy lymphoblasts, and to determine if altered mitochondrial dynamics provides a possible mechanism underlying the beneficial effects of OEA.

Total levels of DRP1 and DRP1 phosphorylated at the activating Ser616 site did not differ between healthy and BTHS lymphoblasts, and were unaffected by OEA. Interestingly, however, levels of DRP1 phosphorylation at the negative regulatory Ser637 site were significantly elevated, indicating that impaired fission may be a factor underlying the increased size of mitochondria in BTHS lymphoblasts. Regardless, phosphorylation modulation at this site is unlikely to be a critical regulator of treatment-related changes in mitochondrial size observed in the lymphoblasts studied, since OEA did not significantly reduce DRP1 Ser637 phosphorylation in BTHS lymphoblasts, but did reduce mitochondrial size. Although total levels of the mitofusins did not vary significantly among groups studied, significant differences in total levels of the fusion mediator OPA1 were detected in vehicle-treated BTHS lymphoblasts, and these levels were returned to healthy control levels following OEA treatment. It therefore seems likely that decreased fission, together with increased OPA1-mediated fusion, are factors in the abnormal enlargement of mitochondria in BTHS lymphoblasts, and that targeting fusion in particular may offer therapeutic benefit. Although OEA has yet to be shown to have actions on mitochondrial dynamics, it is noteworthy that activation of the OEA receptors PPARα and TRPV1 has previously been shown to regulate mitochondrial size in many cell types including fibroblasts, neuronal cells, and cardiac cells^54–57^. Further studies will be needed to confirm a direct role for these receptors in effects observed in BTHS lymphoblasts.

It should be noted that in healthy lymphoblasts, OEA treatment caused a slight, but significant decrease in the number of cells on day 4, and also a significant decrease in average cristae length per mitochondria. This was not expected, and the mechanism was not apparent. Significant OEA-mediated changes in protein regulators of mitochondrial dynamics were not detected in healthy cells in this study. OEA has been found to potentiate beta-adrenergic mediated signaling in adipose tissue^58^, which is more typically associated with increased cristae networks^59^. Further work will therefore be required to more fully understand these outcomes.

In conclusion, using quantitative analysis, the present study demonstrates that BTHS lymphoblasts have a decreased rate of growth, and enlarged mitochondrial size and density, but decreased parallel cristae length, and no difference in mitochondria numbers compared to healthy matched lymphoblast controls. Treatment with OEA significantly increased the number of BTHS lymphoblasts in culture when grown over 4 days, and improved mitochondrial membrane potentials, although the only morphological factor significantly affected by OEA treatment was a reduction in mitochondrial size to more closely match healthy control lymphoblasts. Notably, these effects occurred without significant changes in cardiolipin content or quality. Analysis of protein regulators of mitochondrial dynamics indicated that both decreased fission (through elevated phosphorylation at Ser637 of DRP1) and increased fusion (through increased levels of OPA1) likely play a role in the increased size of mitochondria in BTHS lymphoblasts. However, only decreased fusion, possibly mediated through decreased levels of OPA1, was implicated as a potential mediator of effects of OEA on reductions in mitochondrial size.

When taken together, these results suggest that therapies targeted at decreasing mitochondrial fusion, or possibly also increasing mitochondrial fission, may have potential benefit in treating BTHS. In this regard, the present study identifies OEA as a possible therapeutic for BTHS. However, it is important to note that OEA did not fully restore the growth deficit of BTHS lymphoblasts. In addition, there are contraindications for the direct use of OEA in the BTHS clinical population. For example, OEA has been shown to increase satiety and reduce feeding behavior in animal models^60,61^, which is a source of concern in BTHS, where decreased appetite may exacerbate already prominent symptoms of growth delay and skeletal myopathy. Nonetheless, results from this work provided novel insights on the modulation of mitochondrial dynamics as a possible treatment target for BTHS, and suggest that modulators of mitochondrial dynamics could provide a source for novel therapeutics.

## Methods

### Materials

OEA and LEA were obtained from Avanti Polar Lipids (Alabaster, AL, USA). Unless otherwise specified, all other chemicals and reagents were purchased from Sigma-Aldrich (St. Louis, MO).

### Cell culture

Epstein-Barr virus-transformed lymphoblast from 5 males with, and 5 males without BTHS, aged 10 and under, were obtained from the Coriell Institute for Medical Sciences, National Institute of General Medical Sciences (NIGMS)—human genetic cell repository. Donor characteristics are described in Table 1. Lymphoblasts were grown and maintained in vent-capped cell culture flasks in Roswell Park Memorial Institute (RPMI-1640) media with 10% fetal bovine serum (FBS) and 1% penicillin–streptomycin (P/S), and routinely passaged at low-density. For all experiments, lymphoblasts were seeded in 25 cm^2^ flasks at an initial density of 1.2 million cells per flask, in 7 mL of RPMI-1640 with 10% charcoal-stripped FBS (Gibco, Waltham, MA, USA) and 1% P/S, and treated with either 1 µM OEA, or vehicle alone (ethanol, 0.1% final concentration). Treated cultures were maintained at 37 °C in 5% CO_2_ for a total of up to 4 days, as indicated.

**Table 1:** Characteristics of individual BTHS patient and healthy donor lymphoblasts obtained from Coriell NIGMS—human genetic cell repository.

Order number	Sex	Age (years)	Type of mutation
GM22192	Male	10	C > T change in exon 3 of TAZ (G4.5) gene, resulting in substitution of cysteine for arginine at codon 94 [Arg94Cyc (R94C)]
GM22194	Male	9	Complete deletion of TAZ (G4.5) gene
GM22193	Male	10	T > A change in exon 6 of TAZ (G4.5) gene, resulting in a premature stop codon [Leu166Ter (L166X)]
GM22150	Male	7	G > A change in exon 2 of the TAZ (G4.5) gene, resulting in a premature stop codon [Trp79Ter (W79X)]
GM22163	Male	9	1 bp deletion (171delA) in exon 2, resulting in a frameshift after amino acid 57 [Gly58AlafsX25]
AG15022	Male	10	Apparently healthy individual
AG14731	Male	8	Apparently healthy individual
AG14948	Male	10	Apparently healthy individual
AG14750	Male	9	Apparently healthy individual
AG14947	Male	10	Apparently healthy individual

### Approval for experiments involving human tissue

Experiments were reviewed and approved by the University of Waterloo Research Ethics Board (ORE# 40551) and all experiments were performed in accordance with approved protocols. The cells were transferred from Coriell Institute for Medical Research NIGMS Human Genetic Cell Repository under a Material Transfer Agreement with the University of Waterloo, with R.E.D. as the Principal Investigator, and informed consent was acquired prior to collection/deposition of the cell lines at the Coriell Institute for Medical Research NIGMS human genetic cell repository. Only non-identifying information publicly available from the Coriell Institute for Medical Research NIGMS human genetic cell repository is shown.

### Cell growth measurements

Lymphoblasts were seeded in 7 mL of media at an initial density of 1.2 million cells per 25 cm^2^ flask. Total cell numbers were measured every 24 h, for a total of four time points (96 h). To perform cell counts, each flask was mixed by gentle pipetting using a 10 mL serological pipet, and then a 10 µL draw was removed and mixed with 30 µL of RPMI media to generate a 1:4 dilution. A 10 µL volume from each diluted sample was added to each of the two sides of a hemocytometer, where the cells were manually counted by a single, blinded observer, following the Trypan Blue-exclusion protocol described by Sigma Aldrich^62^. Cells were counted in triplicate or duplicate, and means were averaged to derive a single measure for each flask at each timepoint. Notably, automated counting was attempted, but found to introduce significant artefact due to similarities between the size of lymphoblasts and air bubbles introduced in mixing and pipetting (data not shown), and therefore manual counts were performed.

### Analysis of mitochondrial membrane potential

Cells were cultured and treated for 4 days according to the protocol for cell growth measurements. On day 4, mitochondrial membrane potential was analyzed using the cationic dye tetramethylrhodamine ethyl ester perchlorate (TMRE, Cayman Chemical, Ann Arbour, Michigan, USA) as previously described^63^. Briefly, a TMRE stock solution was prepared at a concentration of 20 µM in sterile dimethyl sulfoxide and stored at − 20 °C. Healthy and BTHS cell lines were seeded at 105 cells/well, treated with 1 µM OEA, LEA or vehicle (0.1% ethanol (v/v)), and incubated with 200 nM TMRE for 20 min at 37 °C in the dark. Cells were then washed with PBS containing 0.2% BSA (w/v), and resuspended in a total volume of 100 µL PBS with 0.2% BSA. TMRE fluorescence was recorded at 549/575 nm using a BioTek Synergy H1 Hybrid Multi-Mode Microplate reader.

### Gas chromatography analysis

Cells were cultured and treated for 4 days according to the protocol for cell growth measurements. Total lipids were extracted using a modified Bligh and Dyer lipid extraction^64^. Briefly, samples were mixed with 0.25 mL of chloroform and 0.25 mL of ddH_2_O, vortexed, then centrifuged at 1000×*g* for 5 min to separate the aqueous and organic phases. The organic phase was extracted and dried under a stream of N_2_. Individual phospholipid species were separated by thin-layer chromatography (Silica Gel Hf, 20 × 20 cm, 250 μM; Analtech Inc., Cole-Parmer Canada, Montreal, Quebec) using a chloroform:methanol:2-propanol:0.25% KCl:trimethylamine solvent system (30:9:25:6:18, v/v/v/v/v) as adopted from Bradley et al.^65^. Briefly, phospholipid bands were visualized by UV illumination after spraying with 0.1% 2,7-dichlorofluorescin in methanol (w/v), and identified and scraped based on comparison with cardiolipin and MLCL standards (Avanti Polar Lipids, Millipore Sigma, Mississauga, Ontario, Canada). In preparation for gas chromatography (GC), the fatty acyl species within cardiolipin and MLCL were derivatized to fatty acyl methyl esters by a transesterification method using 14% boron trifluoride in methanol and hexane (Thermo Scientific, Bellfonte PA), containing docosatrienoic acid (22:3n-3) ethyl ester, as an internal standard (Nu-Check Prep, Elysian MN). This was then heated at 95 °C for 1 h. Following centrifugation at 3000× rpm for 5 min, the top layer was transferred to a fresh tube, dried under a N_2_ stream, and resuspended in 65 µL of heptane. As described previously, analysis using gas chromatography with flame ionization detection was performed using the Agilent 7890A gas chromatograph equipped with a DB-FFAP 15 m × 0.10 mm injected dose × 0.10 μm film thickness nitroterephthalic acid-modified polyethylene glycol capillary column (J&W Scientific/Agilent Technologies, Mississauga, Ontario, Canada) with hydrogen as the carrier gas^65^. Briefly, 1 μL of samples were introduced by an Agilent 7693A autosampler into the injector and heated to a temperature of 250 °C with a split ratio of 50:1. Initial temperature was 150 °C with a 0.25-min hold followed by a 35 °C/min ramp to 200 °C, a 1 °C/min ramp to 211 °C, and then a 80 °C/min ramp up to 245 °C with a 4-min hold at the end. The flame ionization detector temperature was set at 300 °C with air and nitrogen make-up gas flow rates of 300 and 10 mL/min, respectively, sampled at a frequency of 50 Hz. The fatty acyl composition was expressed as both concentrations (μg fatty acids per mg of protein) and relative weight percentages (as a percentage of total fatty acid mass analyzed), and the total CL content was calculated based on the total mass of all CL fatty acyl species within each sample.

### Transmission electron microscopy (TEM) analysis

The protocol utilized was adapted from Acehan et al.^8^. BTHS and healthy lymphoblasts treated with or without OEA were harvested on day 4 after growth and treatment according to the protocol for cell growth measurements into 50 mL Falcon tubes and centrifugated at 1000×*g* for 5 min. The supernatant was removed, and the cells were washed three times with 1 mL of 1× PBS in a series of centrifugation (1000×*g* for 5 min) and resuspension steps. Following the final wash, the sample pellet was fixed in 1 mL of 2.5% glutaraldehyde solution (Polysciences Inc, Warrington, PA) in 1× PBS, and then stained for 1 h in a 0.5% osmium tetroxide solution (Electron Microscopy Sciences, Hatfield, PA) in 1× PBS. Samples then underwent a series of graded dehydration steps using 20% acetone for 10 min, 50% acetone for 10 min, 70% acetone for 20 min, 90% acetone for 20 min, and finally 100% acetone for 20 min. Following the dehydration, each sample was embedded onto an Epon-Araldite (E/A) resin (Canemco-Marivac, Quebec, Canada) (DDSA liquid 30.0 g, Araldite 502 11.1 g, TAAB embedding resin 15.5 g, DMP 30 1.4 g), and ultrathin sections (~ 60–90 nm) were sliced from the resin and collected onto a carbon-coated grid. Images were taken using a Phillips CM10 Transmission Electron Microscope.

Analysis was performed using ImageJ software (version 1.8.0_112), with added macros provided by Springer Protocols (2019). An average of 53.0 ± 10.3 mitochondrial cross-sections were analyzed per cell line to create a single average value per cell line, where each individual line was treated as a biological replicate, and therefore measures are n = 3. The average number of mitochondria per cell was normalized to the size of the nucleus to account for differences in sectioning areas analyzed between cells. Shape was analyzed using the macro ‘circularity tool’, such that a value of one indicates a perfect circle and a value approaching 0 indicates an increasingly elongated shape. Average cristae length was determined based on the sum of the total cristae length per cell, divided by the total number of mitochondria. Crucially, only cristae that are parallel in nature, and are attached to the inner boundary membrane are included in the analysis. Mitochondrial size was calculated based on the average area occupied by individual mitochondria within cells in µm^2^. The number of enlarged mitochondria is based on a cut-off value, where the average number of mitochondria > 1 µm^2^ per cell line were recorded. Finally, mitochondrial density was estimated by multiplying the average number of mitochondria per cell (normalized to the size of the nucleus) times the average size of a mitochondrion in that cell line.

### Western blot analysis

Western blot analysis was performed as previously described, with minor modifications. In brief, cells were grown according to the protocol for cell growth measurements and harvested on day 4 into 50 mL Falcon tubes and centrifuged at 1000×*g* for 5 min. The supernatant was discarded, and the samples were washed an additional three times with 1× PBS. Following the final wash, protein lysates were prepared by resuspending the cell pellet in RIPA buffer (50 mM Tris–HCL, pH 8.0; 150 mM NaCl, 1% Nonidet P-40, 0.5% sodium deoxycholate, and 0.1% SDS with 10 µL/mL of protease/phosphatase inhibitor cocktail (Cell Signaling, Beverly, MA), and protein concentrations were determined using bicinchoninic acid solution. Samples were mixed with 3.33 µL of 6× Laemmeli Buffer (125 mM Tris–HCl, pH 6.8, 20% glycerol (v/v), 4% SDS (w/v), 10% 2-mercaptoethanol (v/v), and 0.05% bromophenol blue) and heated to 95 °C for 5 min, then electrophoresed through 12% SDS-PAGE TGX Stain-Free™ FastCast™ gels (Bio-Rad Canada, Mississauga, Ontario, Canada) at 120 V for 1 h, and transferred onto nitrocellulose membranes using a Bio-Rad Trans-Blot Turbo system (Bio-Rad Canada), set at 25 V for 30 min. Transferred membranes were blocked with 5% bovine serum albumin (BSA) in TBST (50 mM Tris–HCl, pH 7.4, 150 mM NaCl, 0.1% Tween-20) for 1 h at room temperature, then incubated overnight at 2 °C in TBST containing, 5% BSA and primary antibodies (1:1000 dilution) directed against OPA1, MFN1, MFN2, p-DRP1 S616, and p-DRP1 s637 (Cell Signaling Technology, Whitby, Ontario, Canada). The following day, the membranes were washed three times with TBST, incubated with TBST containing horseradish peroxidase (HRP)-conjugated secondary antibodies (Cell Signaling Technology) at 1:2000 dilutions and 5% skim-milk/BSA for 1 h at room temperature, then washed again three times for 10 min per wash with TBST. Membranes were rinsed with 1 mL of LumiGLO (Cell Signaling Technology) for 1 min, followed by the detection of bands using a ChemiDOC Touch Imaging System (Bio-Rad Canada). Protein bands were normalized to total loaded protein, imaged by fluorescence-activation of the TGX Stain-Free gel under UV illumination using Image Lab software (Bio-Rad Canada), to determine relative protein expression levels.

### Statistical analysis

Data are expressed as ± S.E.M, or as individual values. Repeated measures two-way analysis of variance (ANOVA) with multiple comparisons using Sidak’s post-hoc test was used to analyze for the effects of treatment within cell types, and effects of cell types within each treatment during time-course analyses. Differences between groups for measures of cell numbers at each timepoint, mitochondrial membrane potential, mitochondrial morphology measures, and immunoblot quantitative analyses were assessed using two-way ANOVA with Sidak’s post-hoc test. Difference between groups for cardiolipin measures were assessed by two-way ANOVA with Newman–Keul’s post-hoc test. Effects of treatment on cell numbers within groups of individual donors were assessed by Student’s paired t-test. Significant differences were accepted at P < 0.05.

## Supplementary Information

Supplementary Figures.

## Data Availability

The datasets generated during and/or analysed during the current study are available from the corresponding author on reasonable request.
