# Supplementary material for: N-oleoylethanolamide treatment of lymphoblasts deficient in Tafazzin improves cell growth and mitochondrial morphology and dynamics
Source: Sci Rep. 2022 Jun 8;12:9466. doi: 10.1038/s41598-022-13463-z (PMC9178007; doi:10.1038/s41598-022-13463-z)
Supplement: Supplementary file 1 — Supplementary Figures. [file 41598_2022_13463_MOESM1_ESM.pdf]

Supplementary Figures

N-oleoylethanolamide Improves Cell Number Expansion and Mitochondrial Dynamics of Lymphoblasts Deficient in Tafazzin

John Z. Chan[1], Maria Fernanda Fernandes[1], Klaudia E. Steckel[1], Ryan M. Bradley[1], Ashkan Hashemi[1], Mishi R. Groh[2], German Sciaini[3], Ken D. Stark[1], and Robin E. Duncan[1, *]

[1]University of Waterloo, Department of Kinesiology and Health Sciences, BMH 1044, Faculty of Health, Waterloo, N2L 3G1, Canada

[2]University of Waterloo, Department of Biology, Faculty of Science, Waterloo, N2L 3G1, Canada

[3]University of Waterloo, Department of Chemistry, Faculty of Science, Waterloo, N2L 3G1, Canada

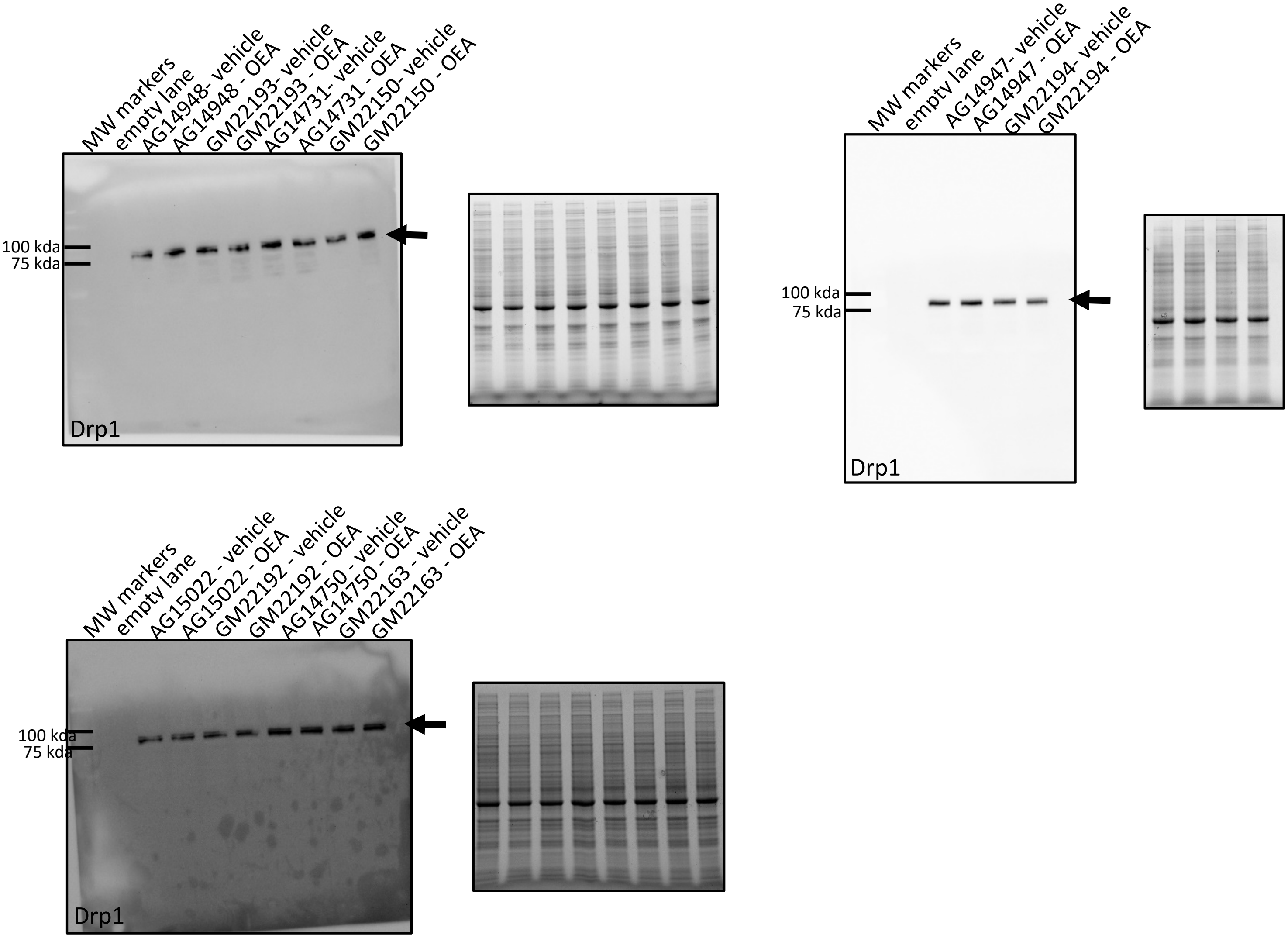

Supplementary Figure 1. Full-length/uncropped/unfiltered immunoblots of DRP1 (~78-82 kDa) in five individual healthy and BTHS lymphoblasts donors treated with vehicle or 1 μM OEA as indicated above the image. Arrows indicate the DRP1 band. Total protein loading for each gel was visualized by UV-imaging of stain-free gels (Bio-Rad Canada, Mississauga, Ontario, Canada), and those images are shown in the panel to the right of each immunoblot.

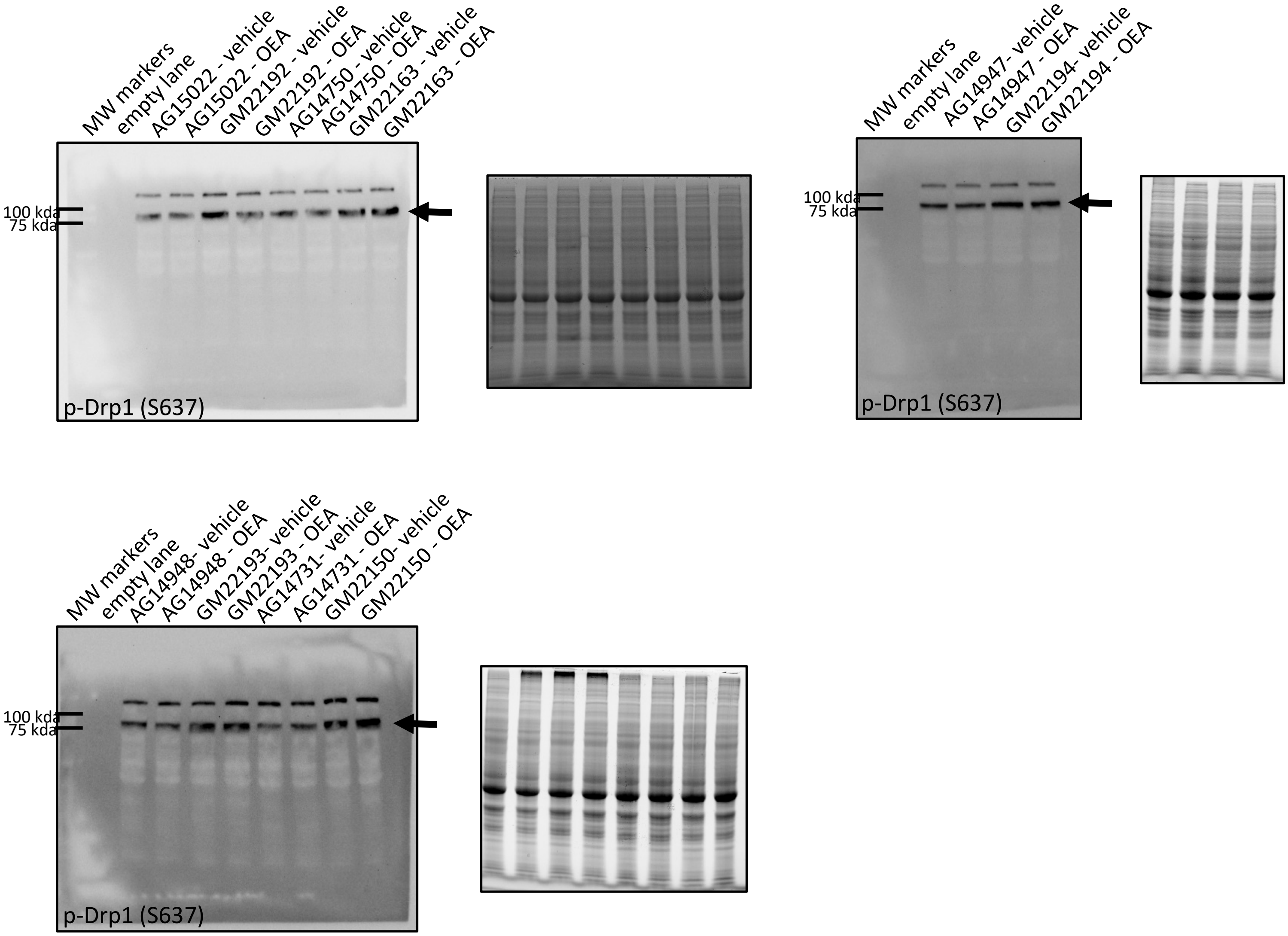

Supplementary Figure 2. Full-length/uncropped/unfiltered blots of p-Drp1 (S637) (~78 - 82 kDa) in five individual healthy and BTHS lymphoblasts donors treated with vehicle or 1 μM OEA as indicated above the image. Arrows indicate the p-Drp1 (S637) band. Total protein loading for each gel was visualized by UV-imaging of stain-free gels (Bio-Rad Canada, Mississauga, Ontario, Canada), and those images are shown in the panel to the right of each immunoblot.

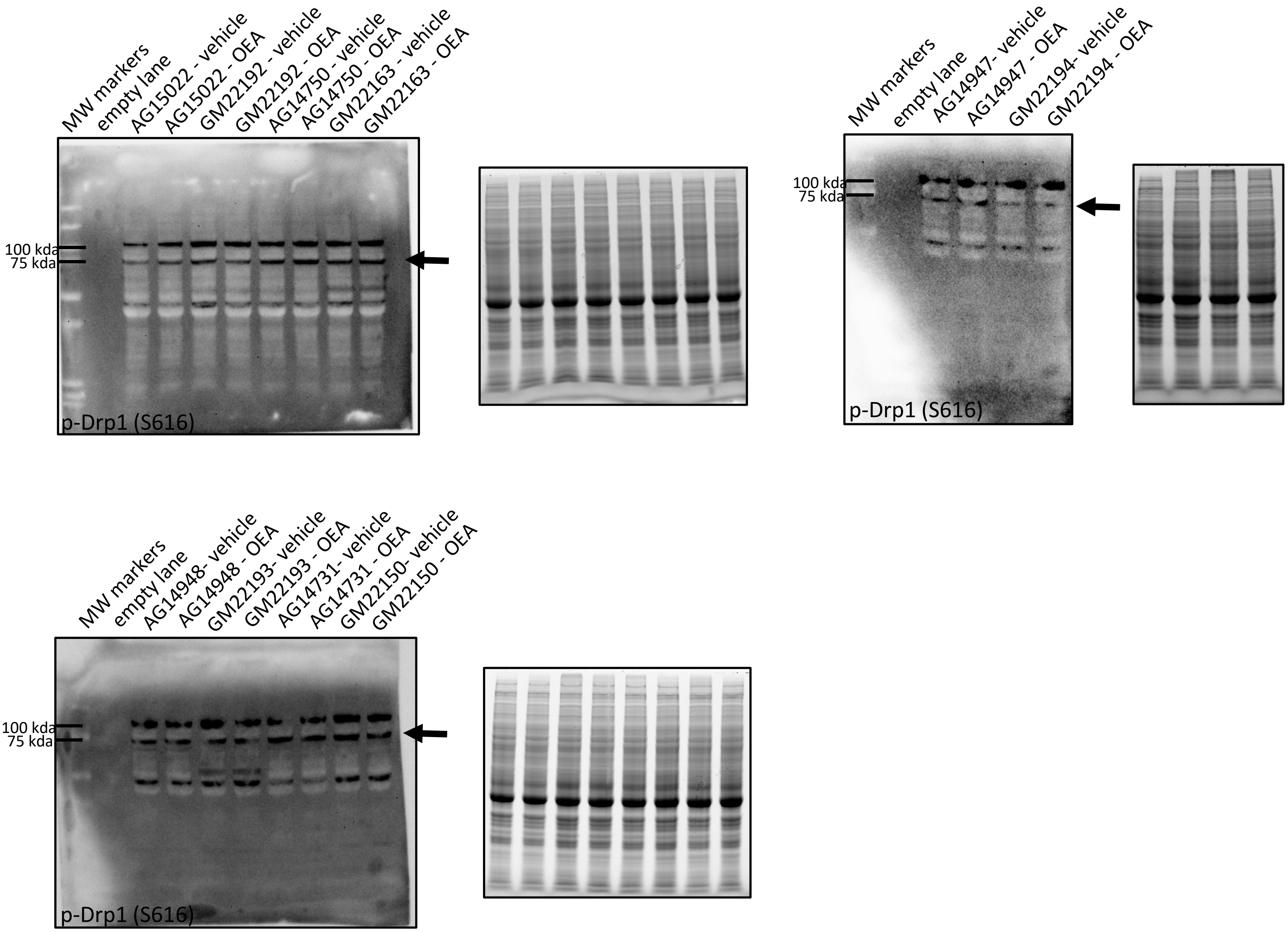

Supplementary Figure 3. Full-length/uncropped/unfiltered blots of p-Drp1 (S616) (~78 - 82 kDa) in five individual healthy and BTHS lymphoblasts donors treated with vehicle or 1 μM OEA as indicated above the image. Arrows indicate the p-Drp1 (S616) band. Total protein loading for each gel was visualized by UV-imaging of stain-free gels (Bio-Rad Canada, Mississauga, Ontario, Canada), and those images are shown in the panel to the right of each immunoblot.

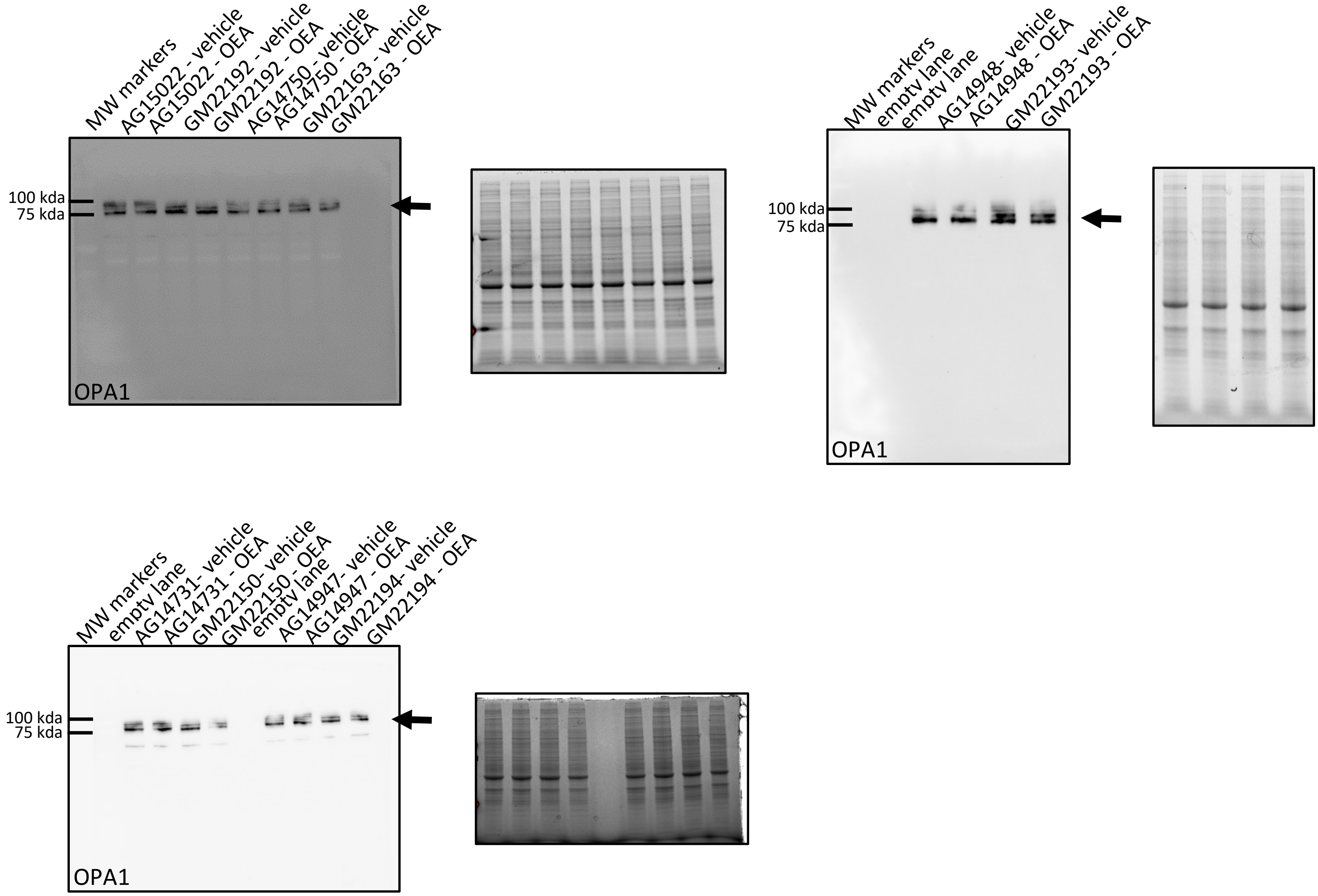

Supplementary Figure 4. Full-length/uncropped/unfiltered blots of OPA1 (~80 - 100 kDa) in five individual healthy and BTHS lymphoblasts donors treated with vehicle or 1 μM OEA as indicated above the image. Arrows indicate the OPA1 band. Total protein loading for each gel was visualized by UV-imaging of stain-free gels (Bio-Rad Canada, Mississauga, Ontario, Canada), and those images are shown in the panel to the right of each immunoblot.

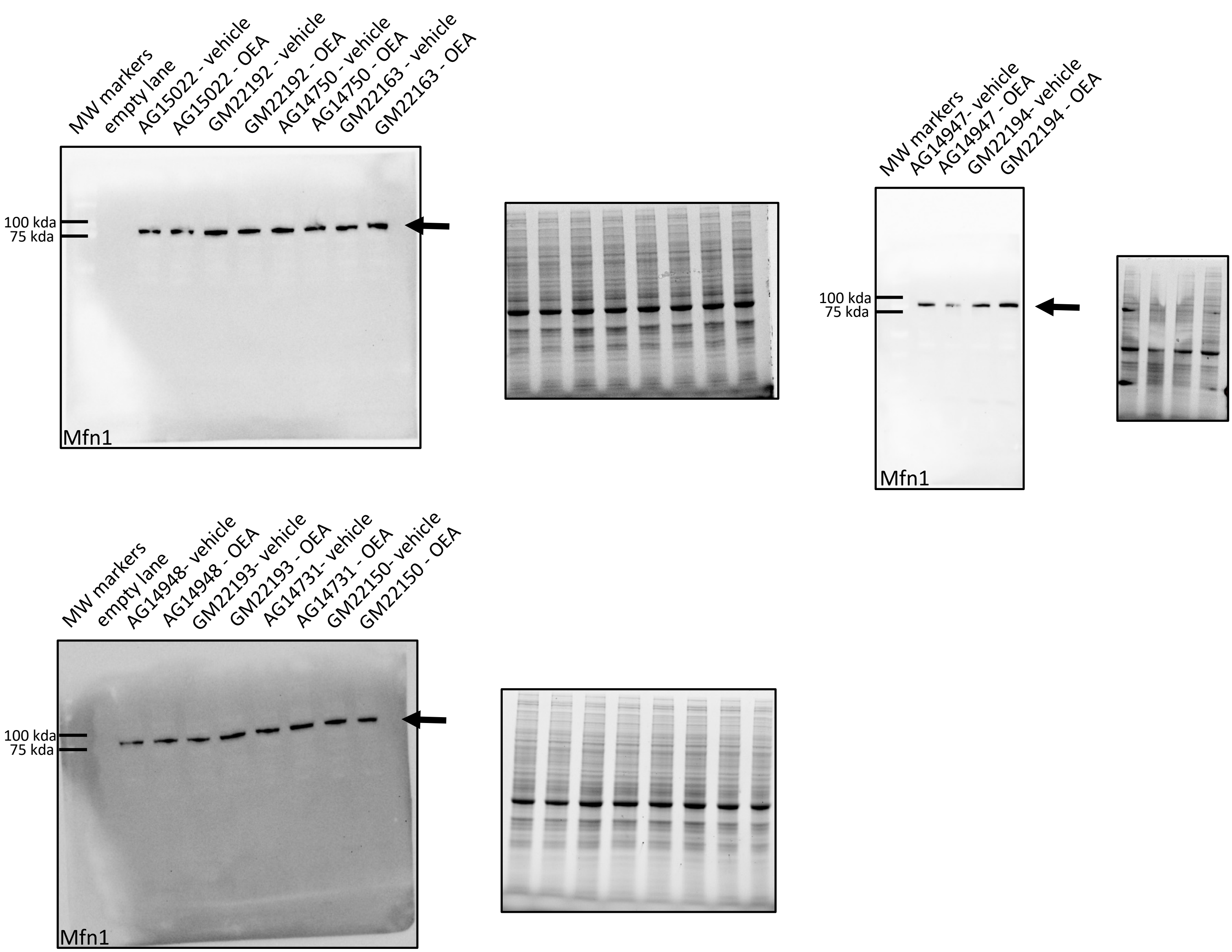

Supplementary Figure 5. Full-length/uncropped/unfiltered blots of Mfn1 (~82 kDa) in five individual healthy and BTHS lymphoblasts donors treated with vehicle or 1 μM OEA as indicated above the image. Arrows indicate the Mfn1 band. Total protein loading for each gel was visualized by UV-imaging of stain-free gels (Bio-Rad Canada, Mississauga, Ontario, Canada), and those images are shown in the panel to the right of each immunoblot.

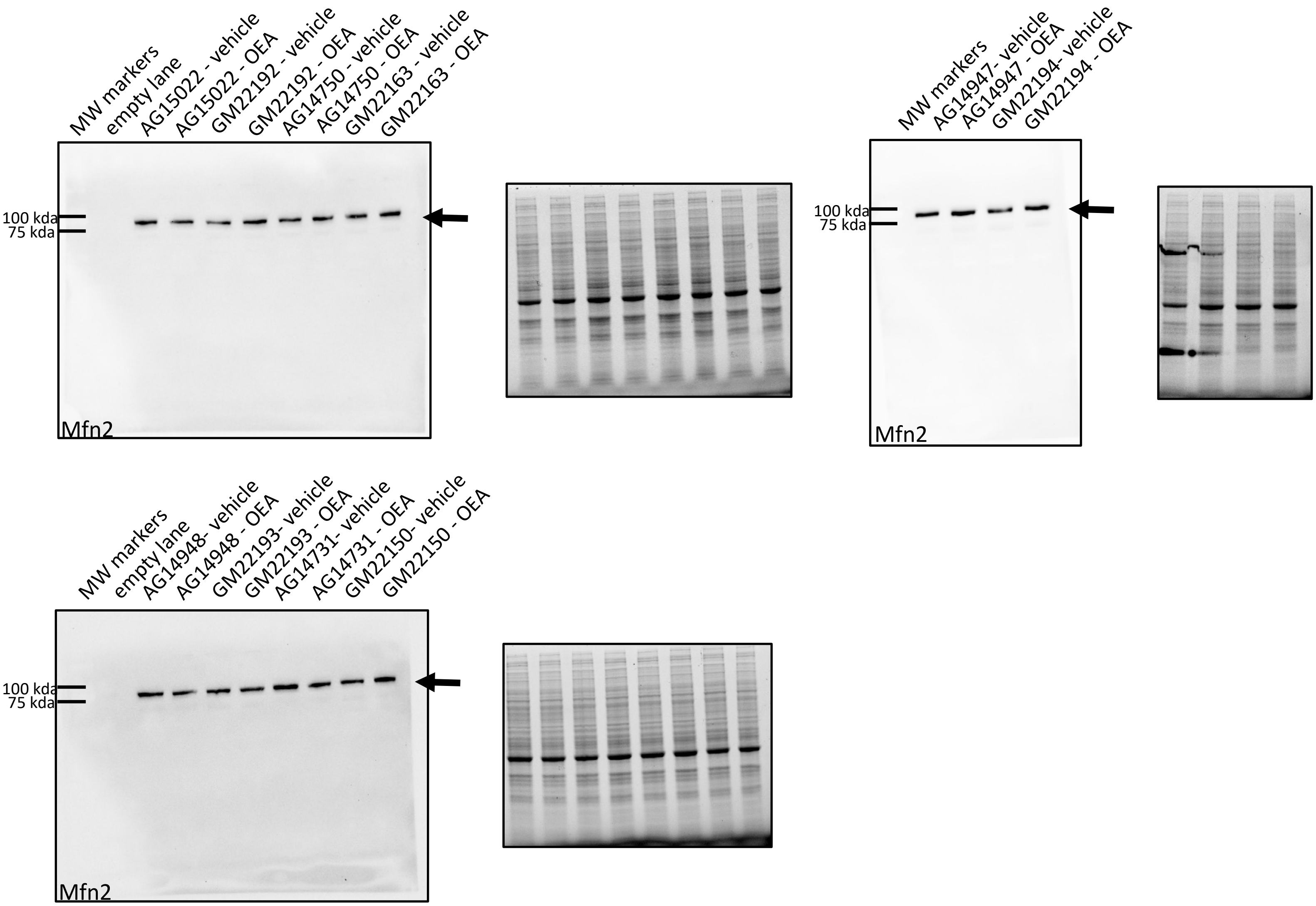

Supplementary Figure 6. Full-length/uncropped/unfiltered blots of Mfn2 (~80 kDa) in five individual healthy and BTHS lymphoblasts donors treated with vehicle or 1 μM OEA as indicated above the image. Arrows indicate the Mfn2 band. Total protein loading for each gel was visualized by UV-imaging of stain-free gels (Bio-Rad Canada, Mississauga, Ontario, Canada), and those images are shown in the panel to the right of each immunoblot.
